# Divided Countries, Divided Mind 1: Psycho-Social Issues in Adaptation Problems of North Korean Defectors

**DOI:** 10.4306/pi.2008.5.1.1

**Published:** 2008-03-31

**Authors:** Sung Kil Min

**Affiliations:** Department of Psychiatry, Yonsei University College of Medicine, Seoul, Korea.

**Keywords:** North Korean defectors, Korean unification, Culture, Personality development, Adaptation, Mental health

## Abstract

A review of studies on the adaptation problems of North Korean defectors in South Korean society and studies of people's adaptation to political and cultural changes in other countries suggests that similar adaptation problems may occur in the process of and after unification. Defectors have various adaptation problems and some of them have psychiatric disorders such as depression and post-traumatic stress disorder (PTSD). The reasons for this were revealed to be the difference in the culture and personality between South and North Korea, which have developed for the last 60 years without any communication with each other, in spite of their common racial and cultural heritage. Economic factors including the lack of skills and knowledge for working at industrialized and competitive society like South Korean society, also aggravate the severity of such adaptation problems. Research on defectors' adaptation problems and on the differences in the culture and mentality between North and South Korea can provide useful information on what kinds of problems may arise during the process of and after unification and what should be done to achieve mutual adaptation and harmonious and peaceful unification.

## Introduction

In the process of Korean unification, the political, geographical, economic and social aspects are all considered important. However, the "unification of the people", not the elite, will be the most important aspect, because the ultimate purpose of unification is the happiness of the people, who will forge a successful and complete unification.^1^ Unification should not be the cause of new conflicts between groups of South and North Koreans, but should secure an improvement in their quality of life and foster health in the long term. If unification were to bring frustration, conflicts and consequently unhappiness to the very people who seek to bring unity, then unification might not be in their best interest. However, the unification of the people will not be simple and easy, even though both North and South Koreans have strongly and enthusiastically held out the hope of reunification for a long time. The main reason for this difficulty is likely to be the difference in the two people's mindsets (mentality or personality) which has developed based on their different cultural experiences. Despite their common cultural heritage, North and South Korea have supported different and even starkly contrasting social systems over the last 60 years. Sixty long years is sufficient time for shaping unique personalities of the people in the two Koreas based upon their life experiences from all the way back to childhood. When people with a different personality and culture come to live together, conflicts tend to develop. Sometimes, such conflicts can be in the form of the so-called culture shock, which, if not tackled early on or left unresolved, may induce long-term complications or even re-separation.^2^

The psychosocial issues of concern in Korean unification were first addressed through studies on the adaptation problems of North Korean defectors in South Korea.^3^,^4^ Currently, they are major subjects who can provide information on the mentality of North Koreans and the everyday culture of current North Korean society. Their adaptation problems in free South Korean society represent a good laboratory situation in which the effect of their different culture on their life, mentality and mental health can be studied.

### Possible contribution of psychiatry

The issue of defector adaptation is a challenge to Korean psychiatrists, because they are professionals dealing with the human mind, adaptation and the treatment of mental health problems. The issues of migration, culture shock and social integration are not only research subjects of social psychiatry, but also constitute the preliminary process of Korean unification. From such research and experimental results, we can gain useful knowledge on the "people's unification" of Korea. The principles of psychotherapy for conflict resolution, including dialogue, understanding, healing and reconciliation are well suited to the process of unification.

In this regard, preliminary studies are essential for predicting, minimizing, avoiding and overcoming possible psycho-social conflicts, in order to achieve smooth, peaceful and harmonious unification. These studies may include research into the adaptation problems of North Korean defectors (NK defectors) in South Korean society, and lessons can also be learned from similar adaptation problems which have arisen in the unification process of other countries or in culturally and politically changing societies.

### Migration, culture and mental health

Problems similar to those faced by NK defectors may develop in people who previously lived in communist countries and have now begun to live in capitalistic countries. These problems related to political changes are basically the same as the adaptation problems of those who have migrated into different cultures or who are experiencing social changes. It has been well studied that bio-psycho-social stress related to a new society, new culture, new custom, new lifestyle and new ideology (value system) may induce adaptation problems or acculturation problems.^4^-^7^ Immigrants have to change everything to survive in a new culture while suffering from anxiety. These states are well represented by the term, "culture shock".

Similar psychosocial adaptation problems following trans-cultural migration have been observed. Hur and Kim^8^ reported on the adaptation of Koreans who had immigrated to the USA, suggesting that the mental health of Korean Americans was dependent on the interaction between their Americanization and ethnic attachment. As for Koreans, Lee et al.^9^ reported on the adaptation problems and poor quality of life in Korean-Chinese laborers in South Korea. Lin et al.^10^ reported the increased severity of the psychotic state and psychosomatic symptoms in Vietnamese refugees in the USA. Bauer and Priebe (1994) reported on the psychopathology and long-term adjustment of 122 refugees from East Germany and indicated that most of them had gone through traumatic experiences such as imprisonment, repression by the state authorities, flight to other East European countries and degradation of their job situation prior to their flight and that the refugees complained of sleep disturbances, nervousness, headache, sadness, repeated crying, sweating, aggressiveness and exhaustion. Most of them were diagnosed as having depressive disorders, anxiety disorders, and adjustment disorders. Yet, eventually they were able to successfully adapt to West German society. Ebata et al.^11^ reported on Japanese war orphans, who had been left in China after WW II and adopted by Chinese parents and who grew up as Chinese. After four decades, many of them returned to live in Japan and experienced difficult trans-cultural adjustment. Anxiety related to assimilating into their culture of origin contributed to their psychological condition.

Especially, migration due to political reasons related with repression, threats, torture, imprisonment, expulsion or exile are known to cause serious psychiatric sequelae.^12^

These studies are comparable to similar studies on NK defectors in South Korean society, which will be described later. Similar problems were found in the lives of NK defectors in South Korean society. Also, it may be speculated that similar mutual adaptation problems will arise between the two groups of Korean people during and after the unification process between North and South Korea.

## Adaptation Problems of North Korean Defectors in South Korea

As the number of defectors from North Korea to South Korea has increased, the various adaptation problems experienced by the defectors have increased as well. These adaptation problems and mental health issues were addressed for the first time by Min and Jeon.^1^ Since then, the author and his colleagues have studied, in series, the lives of North Korean defectors in South Korean society and their psychosocial adaptation problems over the last 20 years.^12^-^30^ Similar studies have been conducted by other social and political scientists.^31^-^40^ These studies have been conducted to find out what kind of adaptation problems the defectors suffered from and to predict harmonious ways to go beyond mere political or geographical unification in order to bring about the "unification of the people".^1^

### Historical review of defection from North Korea

There have been three waves of defection from North to South Korea since the division of the country. The first wave was just before and after the North Korean government was established in 1948. Many people who did not agree with communism escaped from North Korea to South Korea at this time, while others escaped during the Korean War (1950-1953). In total, about 3 million people came to the South. They were generally welcomed by South Koreans and adapted very well.

After the ceasefire in 1953 until 1995, about 100 defectors came to the South for political reasons, either by passing through the demilitarized zone (DMZ), coming by sea and even by air. They were classified by North Korea as criminals. According to Lee^33^ in the 1960s, more than 70% of the defectors said that they fled for political reasons, such as their "quest for freedom", "longing for South Korea" or "discontentment with Communism." According to a National Unification Board 1994 survey of 209 defectors, most of them cited personal motives, including "ill treatment" by the regime or having a "faulty background".

The third wave began with the great famine which gripped North Korea around 1995-1998. Hungry North Koreans near the border between North Korea and China began to cross over into Manchuria in search of food. When some of them found a way to go to South Korea, other defectors began to join in. Nongovernmental organization (NGO) volunteers from South Korea began to go over to Manchuria to help defectors come to South Korea. Others escaped to 3^rd^ countries such as Mongolia, Russia or South East Asian countries in order to contact the South Korean embassy. After the end of the famine, many North Koreans continued to defect for many other reasons, including to search for a better life, to escape from human rights violations or to avoid punishment. The number of defectors has increased recently. As of 2007, more than 10,000 defectors have entered South Korea, and it is likely that the number of defectors will increase in the future according to the economic or political condition of North Korea. Recently, families have been defecting in staggered sequences after years of preparation.^34^ Also, nowadays more females than males are defecting.

#### Support Activities and Research

In the early period, NK defectors were cared for by government officers of the national information institute while they were being investigated. Later, the police inherited the task of protection in the community where the defectors begin to reside. From this period, NGO volunteer helpers began providing support activities, but not all of them were familiar with this kind support activity. Their activities were personal, simple and directive, including helping with routine public activities such as buying things, using the transport system, performing administrative tasks at government offices or banks, providing money or food, and inviting defectors to their homes.

Sooner or later, their adaptation problems in practical life began to be noticed by their helpers. These helpers began to feel their limitation in coping with the defectors' various adaptation problems, especially their mental health problems. At this point, psychiatrists and other mental health professionals began to take an interest in their adaptation problems and in helping them. Research on the adaptation problems and mental health problems of NK defectors and the reactions of helpers had begun.^1^

Based on these studies, support activities have improved gradually and have been systematized together with NGO activities and governmental activities. One of the results of these integrated activities, Hanawon, a national education institute providing the defectors with education about this new society and new culture along with health care for 2 months before their release into society, was established in 1998, when integrated services began to be systematically provided.

Nowadays, not only NGOs but also government organizations (community mental health centers and local welfare centers) are helping them to adapt. Universities, government institutes (e.g. the National Institute for Unification) and government-supporting research institutes (e.g. The Association for North Korean Migrants Studies and Database Center for North Korean Human Rights) and private institutes are conducting various studies on defectors and their adaptation.

Recently, local government and community mental health centers managed by local government began to support defectors in their community. This program is probably the first and most systemic approach by mental health professionals, which provides service and education programs for both defectors and their helpers.

### Shadow of trauma

#### While They Were in North Korea

Jeon et al.^25^ reported on the traumatic experiences that 258 defectors had while they were in North Korea. Their major traumatic experiences were physical, politico-ideological or family-related. Of the 258 defectors, 97.4% were traumatized by watching public executions (killings), 81.3% by watching helplessly their family dying of starvation, 71.2% by watching violent beatings, 38.9% by watching torture, 20.9% by experiencing torture and 2.6% by being raped. Other traumatic experiences included watching executions due to political mistakes, helplessness in the face of their family's illness, anger due to "sungbun", tension related to being suspected for political reasons, lack of food, natural disasters (e.g. floods), being the target of political denunciation or criticism, the suicide of family members, and cold weather. They diagnosed post-traumatic stress disorder (PTSD) in 29.5% of the subjects, compared with the prevalence rate of 1-3% in the general population in South Korea.

#### During Defection

In one article, Newsweek magazine reported that NK defectors arrived in South Korea on the "Underground Railway to Seoul", with the help of NGO volunteer helpers, after crossing the desert, jungle, minefields and wire-entanglements at the risk of their lives.

Lee et al.^35^ reported that 93% of 170 defectors interviewed in China suffered from lack of food and water during their defection, 89% from lack of treatment of their illness, 85% from the unnatural death of family members, 85% from watching death occur, and 40% from torture. Torture included beating, sleep deprivation, starving, forced posture, and electric shocks. PTSD was found in 56%, anxiety state in 89%, and depression in 81%.

Jeon et al.^25^ reported on the traumatic events during defection in 258 defectors which included starvation, physical or sexual abuse, labor exploitation, human trafficking, watching helplessly the death of family or friends, fear of being detected, beaten, arrested or shot, and sent back to North Korea by the secret police of North Korea, Chinese security police or border guards, anxiety associated with being in a strange country, separation from family, physical or sexual abuse, and panic state after becoming aware of the betrayal of their guide on whom they were totally dependent. They also suffered from feelings of guilt for having betrayed their fatherland and leaving their family at risk of persecution.

Malnutrition, anemia, pain syndrome and many other physical and mental disorders^29^ and short stature reflect how hard a life they had and how serious the distress they experienced previously while they were in North Korea and during their defection.

### On arriving at South Korea

On arriving in Seoul, NK defectors are investigated by the intelligence services, and then they are transferred to Hanawon. Previously, they were released into society directly from the intelligence institute after receiving a brief education about South Korea. They are released from Hanawon into society with financial support and housing. In the community, they are under the protection of the local police force for at least two years, who are responsible for not only receiving regular reports from defectors in the district, but also for supporting, guiding and protecting them. Starting from their Hanawon period, NGO volunteers begin to help them physically and psychologically including with their social life. Recently, not only NGOs, but also government organizations have begun to help them with their adaptation.

### Early "culture shock"

#### Surprise of New Culture

When defectors arrive in Seoul, they are surprised to see the extent of the real development of South Korea, which is manifested in the form of a wealthy city full of cars and high-rise buildings with illumination. Many defectors who came to the south directly through the DMZ even suspected that the whole scene that they used to see while they were in North Korea was staged.^13^

Most North Koreans have lived a very simple life: in North Korea, they have few material possessions, live in small houses or apartments with little privacy, eat simple food, travel infrequently, and have very chaste lifestyles. The high-rise buildings, brilliant lights, many cars and geographical complexity of Seoul are often alarming to defectors.

#### Anxiety and Fear

Defectors often report experiencing severe anxiety sooner or later after arriving in South Korea. They are anxious and uptight about their new world and future life which they do not know anything about. Most defectors recognize their ignorance of basic skills such as using the telephone, taking public transportation, banking, obtaining medical services, and enjoying leisure activities.^13^,^32^ They feel embarrassed that they must learn a whole new set of life skills.

Furthermore, since their childhood in North Korea, they have been taught for a long time during the cold war period to suspect and hate South Korea. They had to identify and report strangers to the government, because they might be South Korean spies. Since spies are treated mercilessly in the North, defectors imagine that they will be suspected of being spies and treated likewise in this potentially hostile environment.^13^ Defectors worry about being identified as people from the North, not only because of their different dialect, but also due to their strange attitudes, clumsiness and unsophisticated behavior. In the early period, defectors are suspicious not only of the Korean CIA, but of South Koreans in general, and even of the NGOs who keep watch over them.^13^,^41^ Both the policemen protecting them and NGO workers have reported that one of the main problems of defectors is their mistrust of South Koreans.^18^,^19^

#### Anger

When NK defectors find South Korea to be wealthy, they feel angry that they have been deceived by North Korea.^13^,^32^ They express their anger against Kim Jong Il, the leader of North Korea, who prohibits the flow of information between North Korea and the outside world.

Those defectors who arrive alone feel anger when South Koreans accuse them of being heartless for leaving their families behind in the North^41^ or suspect them of being criminals who had to escape from North Korea or spies sent by the North Korean government. During their debriefing by intelligence officers, defectors often feel anger about being suspected of being spies.

#### Guilt

Indeed, many defectors who come to the South alone suffer from a feeling of guilt for having left their family behind, who, in many cases, are likely to be punished for the defector's "betrayal" of the fatherland.^13^

Some defectors feel guilty for betraying their former fatherland, as they had been taught that it was their utmost duty to maintain their trust and loyalty toward their country and the Great Leader of their country. In North Korea, even suicide is regarded as betrayal of the country.

In the early period when defectors were provided with money, a house and a job, instead of working, they were frequently asked by the government or NGOs to give a talk in public meetings or churches about their experiences in the North or during their escape from the North. Both their employers and later the defectors themselves were dissatisfied with this kind of anti-communist activity. The audience's main interest was to hear about the curious situation inside North Korea or getting the defectors to come to church. Gradually the defectors' main concern became the reward. The content of the speech tended to be modified according to what the audience wished to hear, which caused some of the defectors to feel distress.

### Long term adaptation

It was found that early trans-cultural adaptation was not necessarily associated with the poor long-term adjustment of defectors. Most NK defectors have been found to adapt successfully to South Korean society through a process of gradual acculturation, probably because of their own active effort to adapt and improvements in the support system.^13^,^25^ Min et al.^21^,^28^ compared a survey of 2001 to a previous survey of 1997, and reported improved quality of life. In these studies, defectors evaluated their subjective quality of life as far better than that while they were in North Korea, and higher than South Koreans did.

Jeon et al.^25^ reported that of a total of 553 defectors, 48% were satisfied with their life in South Korea. They were especially satisfied with the living conditions (62%), including the local environment, housing, family life, and access to medical services (76%). But regarding employment and income, only 29% were satisfied. The level of satisfaction was higher in defectors who came with their family.

#### Overall Adaptation Problems

Nevertheless, some of these defectors, overall 20%, have still been experiencing difficulties,^25^ the most serious being economic difficulties (18.3%), followed by difficulties in employment (12.4%), loneliness (11.7%), prejudice by South Koreans (11.5%), differences in language (8.2%), differences in customs and culture (5.2%), lack of information on South Korea (4.3%), and missing their family members left behind in the North (3.4%). Defectors who came with their family reported serious concerns about their children's education and communication difficulties between generations. Young defectors began to suffer from adaptation problems at school and regarding entrance examinations to university.

#### Language Problems

Even though South and North Koreans speak the same Korean language, defectors have difficulties not only in verbal expression, but also in non-verbal communication. In many cases, different words are used in North and South Korea to convey the same meaning or the same words are associated with different meanings. Some words are also spelled differently. Their distinct North Korean dialect intonations easily reveal that they are from North Korea.^13^

Defectors cannot understand terms of foreign origin (English and Chinese), because the only foreign terms used in North Korea are Russian ones. Chinese characters that are commonly used in South Korea and Japan are not found in North Korea. These linguistic differences cause great difficulty in daily life, for example when buying goods in markets. About 60% of defectors report linguistic difficulties.^32^

#### Economic Difficulties

After they calm down from their early excitement, NK defectors begin to suffer due to unemployment, poor ability to survive in the competitive, capitalist, industrialized and market-oriented society and lack of social-economic support. They are not prepared at all for this new culture.^13^,^19^

In the early period defectors got a job in big companies under government management, but they usually quit their job sooner or later, because they felt that the job did not meet their high expectations for working in big companies. They tend to become overly sensitive about discrimination and feel compelled to leave the job.

In recent times, the government has stopped arranging employment for defectors. After providing a short period of education about living skills, they are released into the society with minimum financial support. For a while, they are supervised to some extent, but soon they have to find normal jobs by themselves or they have to learn elementary occupational skills to find a future job. This new policy of independent living is aggravating the adaptation problems, because defectors have been accustomed to the dependent lifestyle they enjoyed in North Korea.

According to a recent survey,^40^ the proportion of defectors participating in economic activities decreased to 49.3% in 2006 and this rate is lower than that of South Koreans (62.3%), probably due to poor health and being occupied by re-education program. The unemployment rate is also decreasing, probably due to the lack of support of the government. In spite of the increased determination to work of the defectors themselves. It is still 5-8 times higher than that of South Koreans. Women have more difficulty in getting a job than men. Their jobs mostly involve manufacturing and working in small restaurants and, in addition, the stability of their employment has been found to be very low. They spend 500,000-1,000,000 Korean won a month on their living expenses. One third of them were able to save money, while 20% had an average debt of 500 million Korean won.

Yoon and Paek^38^ reported that human resources (previous education in North Korea and South Korea) and social capital (intimacy with South Koreans, duration of living in South Korea and the number of supporting organizations) are significantly important.

#### Frustration With South Koreans' Self-centeredness and Social Vices

In the eyes of defectors, who come from a closed socialist country where keeping faith, sharing or having humanistic affection with their comrades or neighbors are highly appreciated, South Koreans are deemed to be extremely self-centered, selfish, individualistic, aggressive and mercenary.^13^ Defectors initially expected South Koreans to be brothers or "good" people, as they are rich. All too soon, they learn about the grim reality of crimes as well as the discourtesy, impoliteness and even intrusive behaviors frequently found in the streets of Seoul. Some of them are swindled or deceived. Being totally unprepared and unequipped to absorb the realities existing in an industrialized metropolitan city, they feel deceived, disappointed, betrayed or hurt by the selfishness of South Koreans. This makes it difficult for defectors to form genuine friendships with South Koreans.

#### Difficulties in Family Life

In the early period, defectors came to South Korea individually. Now many defectors come to South Korea as a family. Singe defectors or defectors whose spouses remained in the North married other defectors while they were in China or married defectors or South Koreans in South Korea. Accordingly, their family situations are very varied and complicated. Therefore, many and various problems of different complexity have been found in defectors' families. In any case, because of their different culture related to family life, they tend to suffer in South Korea. The problems that arise are related to their familiarity with the previous family culture of North Korea, conflicts between the original family members and new family members from a new marriage, managing money, the generation gap, and family members who were left behind in the North.^37^

#### Difficulties in Relationship With Other Defectors

Generally, it is natural that immigrants should get help from people from the same home country. However, NK defectors experience difficulties in mixing with other defectors. They find that there are differences regarding their attitude to their former father country, life values or money. Therefore, they do not want to expose their past personal history and inner mind, partly because they are sensitive to and fearful of possible criticisms or blame by other defectors. Consequently, they tend to keep some distance between each other and become uninvolved.^18^-^20^ This is one of the unpredicted findings.

Though it would be natural for refugees from the same country to seek each other out, their deep-rooted suspicion and mistrust learned in North Korea prevents them from establishing bonds with other defectors.

#### Confusion in Identity

Many defectors are confused about their identity as citizens of South Korea.^7^ On the one hand, they no longer see themselves as North Koreans, but they still do not believe they are completely South Korean. Loneliness, difficulties in adapting to a new culture and feeling guilty about their families left behind may seriously confuse them. While defectors may hate the social and political system of North Korea and harbor no wish to return, they still hold strong affection for their families, neighbors, friends and hometown that they left behind in the North.^25^ Sometimes, they talk about their fond memories of the life and positive experiences they had in North Korea. Even after many years, defectors often feel angry when South Koreans express hostility towards North Korea. About one third of defectors would prefer that they be called "free North Koreans" or "free immigrants", because of the negative connotations attached to the term "defector".

#### Emotional Life

Fragile pride, tension, anger, depression, helplessness and even persecutory mood are easily found in defectors' emotional response. Some defectors show sudden changes, becoming sullen, when South Koreans criticize North Korea. Defectors look polite, but they may become passive aggressive and even impulsive and violent. Defectors are generally dependent and do not dare to take the initiative.

The cautiousness and suspiciousness, which defectors developed to survive suppression while they were in North Korea, become a habit. In this sense, defectors are repeating their effort to keep their inner emotional reactions inside, so as not to jeopardize their safety even in free South Korean society. These attitudes have resulted in various defense styles^23^,^24^ including withdrawal, control, denial, splitting, isolation and reaction formation.

#### Religious Life

It is generally believed that religious or spiritual life is important for one's well-being. Therefore, it is important to understand how NK defectors interact with religious institutes in South Korea. They tend to have a stronger extrinsic religious orientation due to their previously attained prejudice against religion. While they were in the North, any religious practice was forbidden, not by the constitution but in many indirect ways. Instead, the *juche* ideology (a socialistic ideology of self-determinism), combined with worship of the "Great Leader," constituted the only accepted belief system in North Korea.^42^

Though they do not believe in or understand religion, when they first arrive in the South, defectors attend Christian or other religious meetings in order to show gratitude towards the religious organizations that provided them with assistance.^1^ In a survey, it was found that about 70% of defectors practice a religion, with 61.9% becoming members of Protestant churches, 3.8% the Catholic Church and 2.3% Buddhists.^25^ These figures reflect the efforts of Christian NGOs to help defectors to defect, adapt to life in South Korea and to evangelize Christianity. Defectors who adopted a religion reported that it was helpful in adapting to life in South Korea, because belief gives them peace of mind.^30^

### Quality of life

Their adaptation problems would be finally reflected in subjective quality of life (QOL) of defectors. In a study in 2000 with the World Health Organization (WHO)-QOL Brief Scale, compared to South Koreans, defectors showed better QOL in items of self-esteem, physical appearance, medical services, educational services and religious support, while they suffered more from negative feelings and poor economic life.

In 2003, Min et al.^21^ repeated the study on the subjective QOL of 151 North Korean defectors with the same scale. The defectors were found to be relatively satisfied in terms of their physical health, intellectual functioning, self-esteem, safety, family environment, financial resources, medical and welfare services, physical environment, and the transport system. However, they were not satisfied as regards their positive and negative feelings, mobility, opportunity to acquire new information and skills, and leisure activities. Higher QOL was related to higher income and living with their spouse, and lower QOL was related to older age, living with family, negative life experiences, physical illness, PTSD and depression. However, gender differences, school history in North Korea, experience as a soldier and member of the Labor Party (privileged status in North Korea), duration of living in South Korea, number of education programs received for future job, currently having a job, duration of working, financial support and religious life were not significantly related to the score of QOL.

## Mental Health Problems

### Post-traumatic stress disorder

Many studies have reported that the present adaptation problems of NK defectors are closely related to the emotional trauma they experienced while they were in North Korea and during their escape from North Korea.^26^,^27^,^40^ As a result of these painful experiences, PTSD was found in 56%, anxiety state in 90%, and depression in 81% of defectors in Manchuria.^40^ Among the defectors who had lived in Seoul for 2-3 years, 29.5% were found to have post-traumatic stress disorder. However, Hong et al.^27^ reported in a 3-year follow-up study with the same group that the incidence of PTSD had decreased rapidly to 3%.

### Depression

Recently, Jeon et al.^25^ reported that 16% of 553 defectors had experienced depression. Previously, the depression of defectors was suspected to be related in part to the learned sense of helplessness which they had lived with in North Korea^15^ and in part to the lack of information, professional skills and other abilities required to live in a competitive capitalistic society. The other factors contributing to depression were reported to be loneliness followed by social withdrawal. Guilt associated with their leaving their family behind and suppression of anger seems to aggravate their depression. All of these experiences seem to cause defectors to feel discriminated against and to belong to an inferior class (so-called 2^nd^ class citizens) in South Korean society. Jeon et al.^25^ added past traumatic experiences as important contributing factors.

### Psychosomatic illness

According to Kim et al.,^29^ defectors evaluate their health state as being worse than that of South Koreans. They reported that they were suffering from many chronic diseases. The most frequent were pain syndrome followed by gastrointestinal diseases, anemia and depression. According to the author's personal clinical experiences with defectors and other clinical reports on the illness of defectors, many of them suffer from somatic symptoms related to life stress, anxiety and depression. Common symptoms include back pain, gastrointestinal symptoms, chest oppression, headache, anorexia, disturbing sleep, palpitation, constipation and dizziness.

## Psychology Underlying Adaptation Problems of Defectors

### Coping and defense

Cho et al.^23^,^24^ reported the defense styles which were more frequently adopted by defectors than by South Koreans and related them with their previous socio-cultural experiences. Defectors were found to use active defense and emotion-repression styles more frequently than South Koreans.

Emotional repression seems to be helpful for surviving in North Korean society. This defensive attitude seems to be related to other similar defenses, including inhibition, withdrawal, control, denial, resignation, splitting, isolation and reaction formation. Here again in a free society, they still tend try to keep their inner emotional reactions inside so as not to jeopardize their safety.

The defectors' active defense styles, including task-orientation and prediction, seem to indicate that they are different from North Koreans who did not defect. In reality, they are active, courageous, confident and strong people who took their fate into their hands when they defected from North Korea during which time they had to overcome many obstacles. These character attributes may be partly related to previous education about North Korean virtues such as task-orientation, enduring hard labor and the effort needed to survive desperate socio-economic situations.

These characteristic styles might influence their adaptation in South Korean society in many ways. For example, disciplined and humble attitudes usually give a very good impression to South Koreans, especially the more elderly ones who have been accustomed to traditional Confucian ways of life. Active styles including task-oriented styles are good for finding a job and making the effort required to achieve success, and ultimately to maintain good mental health as well. The task-oriented style and double standards which they learned while they were in North Korea (neglecting their moral duty if necessary and doing anything to survive) might influence positively their adaptation in South Korean society.

However, their passivity, cautiousness, suspiciousness and splitting tendency may interfere with their ability to communicate and relate with their South Korean neighbors. When they experience failure, they tend to withdraw like a lonely wolf. They have difficulties in asking for help from others, try to solve their problems by themselves and endure all the difficulties alone. As they encounter repeated failure when trying to cope alone, their frustration used to be expressed suddenly as anger. Through their long duration of tension in North Korea or even in South Korea, they are sometimes easily provoked into expressing anger explosively. Also, due to their splitting (dichotomizing) tendency, they classify people into two groups, friends and enemies. They become easily suspicious and paranoid and, once they see that something is definitely wrong or feel betrayed, they become impulsively angry or tempestuous or put a stop to the relationship. These unpredictable behaviors give their South Korean friends the impression that defectors are people who cannot be understood and are not reliable.

Defectors show a tendency to somatize their emotional difficulties. They also easily project their problems onto South Koreans or the South Korean government when they encounter ill-treatment and discrimination. Male defectors were found to use more frequently the defense of oral consumption. This may explain why they tend to be dependent on smoking or drinking alcohol.

### Cognitive and behavioral characteristics

#### Way of Thinking

Kim,^41^ a defector himself, suggested that defectors are rigid in their way of thinking and behaving because of their previous uniform education and discipline resulting from the totalitarian ideology. The police protecting defectors complained of their "socialistic" (dependent and demanding) ways of thinking and behavior patterns, which were found to cause them more problems in the long-term.^18^ NGO volunteer workers also reported that 11% of defectors had shown markedly different ways of thinking or different value systems from those of South Koreans; 4% had shown lack of will for independent living and expected lots of help from others, and another 4% had shown a marked tendency to avoid meeting people.^19^

#### Impulsivity

The police protecting the defectors reported that, although their general relationship with defectors was not so bad and had been improving gradually with time, sometimes, the impulsiveness of defectors caused the relationship between them to deteriorate.^18^ This impulsiveness seem to originate not only from the tension and anxiety related to their difficult life in South Korea, but also from the aggressiveness they learned while growing up in North Korea.

#### Social Skills

Defectors do not have sufficient knowledge and skills to live in a free, competitive, capitalistic and industrialized society.^19^,^32^ Defectors generally look different from South Koreans who are living in Seoul. They look poor and boorish, physically short, slim and weak, and are clumsy, shy or tense. They seem to be cautious about communicating due to the different use of language. They seem to avoid close relationships with their South Korean neighbors.

They are often overly sensitive to their inexperience with unfamiliar tasks, their lack of skills and their lack of knowledge of computers and management systems. They are often sensitive to even the age discrepancy between themselves and their South Korean co-workers, probably due to previous concepts of social hierarchy related to age.

When NGO volunteers were asked whether they would employ defectors whom they had helped, 67% answered "yes", but 39% said their role was only to help them. Thirteen percent replied "no", citing the following reasons: the defectors' lack of willingness to work (50%), differences in their way of thinking (20%), and their lack of skills (20%).^19^ However, defectors used to interpret the situation to be a result of unfair discrimination.

A recent report^25^ suggests that the number of defectors who are unemployable due to their lack of skills and ability has decreased through government efforts to provide defectors with improved, systemic, but individualized education programs.

#### Skills of Managing Money

Defectors do not even know how to manage money, e.g. using a bank. They easily fall prey to gambling or even simple fraud or fail to keep or save money. Some of them cannot resist the lure of luxurious living and spend everything.

Defectors have a distorted concept of money and display a unique style of selfishness. Defectors say that money is not important at all in their lives, but in reality they seem to depend on money. They are preoccupied with the various avenues of earning money, are very stingy and try their utmost to save as much money as possible, even employing shrewd methods.^19^ They are convinced that life without money means death in a capitalistic society like South Korea.

Like other double standards, defectors praise South Korea because money can be earned freely through effort, while they criticize South Koreans for using money to solve problems.^32^ This double standard creates difficulties.

### Other influencing factors

#### Positive Factors

The hope for a successful and rich life in a new society, no language problems, shared traditional Confucian culture, common emotional reactivity and strong brotherhood as Koreans are the positive elements for successful adaptation of defectors.^13^ For example, a young male defector may hope to become a successful businessman like his predecessor (Mr. Jung Ju Young, the former president of Hyundai Group). The capitalistic or market oriented social system is attractive to defectors, because it respects the human need for private possession. Defectors try to adapt themselves to such system as fast as possible. Koreans, who are traditionally industrious and eager to learn, will soon master the situation, once they are stimulated and given the chance. Also, their experience of compliance and adaptation to a strict social system while they were in North Korea may help them to adapt to a unified society.

Many NGO volunteer workers also reported that defectors have a number of personality traits or behavior patterns, which may help their adaptation. They have the good virtues of humanistic affection (23%), courteousness and politeness (23%), active and positive attitude (18%), strong will to survive (13%), simple and naive personality (11%) and composure (13%).^19^

#### Negative Factors

Negative factors include worry about discrimination resulting from prejudice, lack of preparatory information, limitation in verbal and non-verbal communication, difficulties in making new relationships with new neighbors, difficulties in finding a new job and feeling that their social status is lower than ever before.

NGO volunteers reported that the most common negative element in defectors was their non-compromising aggressive attitude (22%), but this attitude seemed to improve gradually. Others included their dependence and lack of will for independence (22%), money-oriented selfishness (14%), which worsened gradually, avoidance of interpersonal relationships (12%), male dominance (9%) and their newly formed habit of wasting money (5%). Such reactions may develop during and after unification as well.^19^.

## How to Help Defectors

### Theoretical considerations

For the purpose of helping refugees' mental health, Susser^43^ proposed the "triad model" integrating the agent (e.g. culture shock), host (refugees) and environment. Practically, this means 1) preventing any more shock or trauma, 2) helping refugees to be stronger to cope with shock or trauma (religious help, environmental improvement, providing them with the opportunity to learn about culture, language, social system, family life, economic life with job), 3) continuous and relayed support systems (in the case of NK defectors, support should be started from their arrival at the Korean embassy and continued systematically through to their arrival in South Korea, during their time at Hanawon, during their release into the open society, and extend to their stabilizing period in South Korean society and thereafter) and 4) communitybased support system (recently started for defectors). For NK defectors' adaptation, support system and practice seem to have developed in a heuristic manner according to this triad model.

### Strategies

In early 1996, Kim,^41^ himself a defector, suggested, on the basis of his own successful experiences and the observations of other fellow defectors, the following strategies to help NK defectors adapt to South Korean society: 1) integrated simultaneous assistance by citizens, NGO and government organizations, 2) helping defectors not as people to be protected and needing sympathy, but as citizens who have contributed to a change in North Korea by their defection and can contribute to Korean unification in the future, 3) individualized education programs for acculturation based on the needs and personality characteristics of defectors, 4) providing comfort and healing independent of blood-relation, territoriality and schooling, 5) providing with jobs and financial support by industrial business companies not only in terms of supplying manpower but in terms of increasing harmony between two Koreas. Min^2^ suggested that: 1) Education allowing for better adaptation should be continued until they reach a good level for adaptation. 2) Social support provided by both the government, religious organizations, NGOs, individuals and business companies should be made available in the form of integrated and networked services, with specific professional roles. Among them, employment providing by business companies may be the most important and practical 3) Education and training should be given to South Korean helpers to prevent them from experiencing burn-out.

An examination of the experiences of police officers providing protection^18^ suggested the "humanistic (affectionate)" way and frequent dialogues and contacts (17%) as effective strategies for defectors' adaptation as well as maintaining their positive relationship. Giving them practical help with their daily activities (e.g. performing administrative tasks at a government office or bank in place of the defectors (11%) seemed to be helpful in the early phase, but later was found to make the relationship and adaptation gradually worse). Trying to provide full educational opportunities and advice for living in South Korean society (10%) was found to be very effective. Simply providing money (9%) was found to be a poor strategy.

All activities to help defectors should be integrated into a network of government organizations, civil organizations, religious organizations, individual volunteers and business companies. For activities to be successful, the people who help defectors should be provided with training to prevent their burn-out. These suggestions can also be applied to the unification process.

A recent study^25^ suggested that economic support should be provided according to the individual defector's specific needs, through a process of application, evaluation and contract. All of these proposals and activities can be applied to the unification process.

Yoon and Paek^37^ suggested extended education programs, improving interpersonal communication among defectors and South Koreans, improving and extending their social network, special care for the socially isolated and providing alternative families for orphan defectors.

#### Family Support

Kim^38^ suggested that the defector's family could adapt to the family culture of South Korea through education, modifying their previous concepts (male dominant family culture), legal assistance and community based support for the family.

#### Community Life

Lee^39^ investigated the early adaptation process and adaptation problems of defectors in the community and the influencing factors and suggested systematic networked intervention, case work in integrated form with NGOs and government, networking of resources in the community, and the development of programs to improve their intimacy with residents in the community.

#### Suggestion From Quality of Life Study

A QOL study on NK defectors suggested that, to improve their, they need medical services and mental health services, support for education and recreational life.^23^ Especially, they need education to cope with negative experiences in South Korean society. They need support for religious life and practical social support rather than simple monetary help. Also, the study suggested research on solving familial conflicts and the adaptation of elderly defectors.

### Mental health services

Defectors' mental health problems seem to be related not only to the stress associated with their adaptation to life in South Korea, but also to their past traumatic experiences in North Korea and during the process of defection.

In the early period of helping defectors, the primary concern was their physical health with physical aids including food, housing and jobs. Nowadays, researchers, helpers and government officers are beginning to realize that mental health is as important as physical health for successful adaptation. Until now, counseling for mental health was practiced mainly by religious or NGO volunteers and psychiatric treatment was practiced for selected defector patients with psychiatric disorders by volunteer psychiatrists.

Defector mental health represents a new challenge for psychiatrists and mental health professionals in terms of both research and providing defectors with practical assistance. Mental health professionals have suggested a variety of treatment methods, educational programs, and interventions for defectors.^1^,^2^,^32^ First, special mental health programs and networks are needed for the treatment of PTSD, depression, psychosomatic disorders and other psychiatric disorders.

Nowadays, mental health evaluation and service is practiced from the Hanawon period and many community mental health centers and welfare centers in Seoul and the provinces have just started to provide defectors in their catchment areas with mental health services. The fragmented mental health services of these systems are being networked gradually through systemic education.

#### Stigma and Rarely Seeking Help

Although many North Korean defectors suffer from mental health problems, they lack the concept of mental health as compared to physical health and, consequently, they rarely seek psychiatric help. This failure to seek psychiatric assistance is not because of the inadequacy of South Korean medical services, nor the defectors' unfamiliarity with accessing medical services. In fact, most defectors express a high degree of satisfaction with the medical services available in South Korea.^21^,^25^

The defectors' reluctance and delay in seeking treatment are often due to ignorance of mental health issues and to the stigma attached to psychiatry by both the defectors themselves and most South Korean helpers.

The North Korean government provides no mental health treatment for neurotic conditions such as anxiety, phobia, depression or stress, on the grounds that North Koreans live in a paradise and thus such disorders are groundless. North Korean officials, for example, view suicide as treason against the state, rather than as the result of a depressive disorder. Psychotic patients are often isolated in rural mental institutions (typically called number 49 hospitals), and neurologists not psychiatrists treat emotional problems such as anxiety and depression, which are regarded as a dysfunction of the autonomic nervous system. Therefore, to defectors, seeing a psychiatrist means that he/she is crazy or mad.

### Education

For the prevention and treatment of adaptation problems and mental health problems, education is essential, especially regarding the democratic process, free market economics, practical life-skills, and job training. Education should be systemic, integrated, practical, continuous and individualized, based on the needs and personality characteristics of the defector.

Upon the review of previous studies, the author^2^ suggested a systematized, practical and concrete education program for defectors to allow for their acculturation and adaptation during their protection period. These education programs for adaptation should be continued for a while, even after their release into society until a sufficient level of adaptation is reached. NGO volunteers workers helping defectors also suggested early education for the purpose of promoting democratic or capitalistic thinking and practical education to provide them with skills for managing money as well.^19^ Later, Hanawon became responsible for this early form of individualized and focused education program.

As regards the long term education programs for defectors, Jeon et al.^7^ suggested: education for 1) increasing their internal ability for independent decision-making, 2) overcoming their rigid moral attitude and judgment, 3) overcoming excessive ideology and idealism, and 4) overcoming collective-oriented thinking and dependency. Yoon and Paek^37^ suggested an extended education program based on their research findings on the influences of human resources (previous education in North Korea and South Korea) and social capital (intimacy with South Koreans, duration of living in South Korea and the number of support organizations) on employment.

### Helping the helpers

As the number of defectors increased, more helpers were called on. The number of NGO volunteers has thus increased. Now, there are many NGOs and semi-governmental organizations which are established to help defectors, but they are not yet networked systemically. Still, these NGOs work individually and the helpers work individually too. Some parts of the expense associated with their work are financially supported by the government. However, this support is not sufficient and they still mostly depend on donations from religious organizations, most of these being Christian churches.

The helpers have begun to feel the need for systemic education. However, education programs for helpers have been conducted randomly and not systemically networked. When Hanawon opened based on the suggestion of mental health professions, psychiatrists, clinical psychologists and social workers began to work together and a consensus was formed as to what kind of education is needed for NGO helpers. Recently, research into the burn-out of helpers was conducted, which found that the main etiologies of physical burn-out were excessive duty, lack of professionalism, lack of feeling of achievement. These findings suggested the need to develop healing programs and an incentive system and, to prevent burn-out, reduce their level of duty, strengthen professional job education and develop programs for increasing optimism.^44^

## Conclusion

All of these studies suggest that the adaptation problems of North Korean defectors in South Korean culture are similar to those found in refugees from former communist countries, and seem to be unavoidable. Furthermore, this research provides a new perspective as it provides new information not only on the theoretical influences of ideology and social systems on personality development and mental health, but also on identifying ways of helping people, both practically and clinically, with the psychosocial distress which is characteristic of political indoctrination. In this regard, first of all, encounter and communication between South and North Koreans are essential prerequisites. Then, to promote communication between them, South Koreans have to take the initiative, but also have an empathetic manner.

Studies on the adaptation problems of North Korean defectors in South Korean society in comparison with other cross-cultural studies and the discovery of culture-specific ways to help them are new challenges for psychiatrists and mental health professionals in Korea. For this, research and practice according to medical or psychiatric paradigms would be appropriate. This includes the bio-psycho-social model for health and disease and the study paradigm for concept (definition), epidemiology, etiology, symptoms, diagnosis with differential diagnosis, treatment and its side effects, and prognosis (outcome) of disease. The psychiatric paradigm for conflict solving including communication, understanding, healing and reconciliation can be used not only for the conflicts related to adaptation problems due to cultural differences, but also as research methods.

However, further and deeper systematic researches are needed not only on the different cultures of South and North Korea and the life and personality development of ordinary South and North Koreans, but also on the strategies that can lead to the successful mutual adaptation between NK defectors and South Koreans and of people in a unified Korea.

Starting with the results of small and limited experiments, the steps to the final goal can be built.
